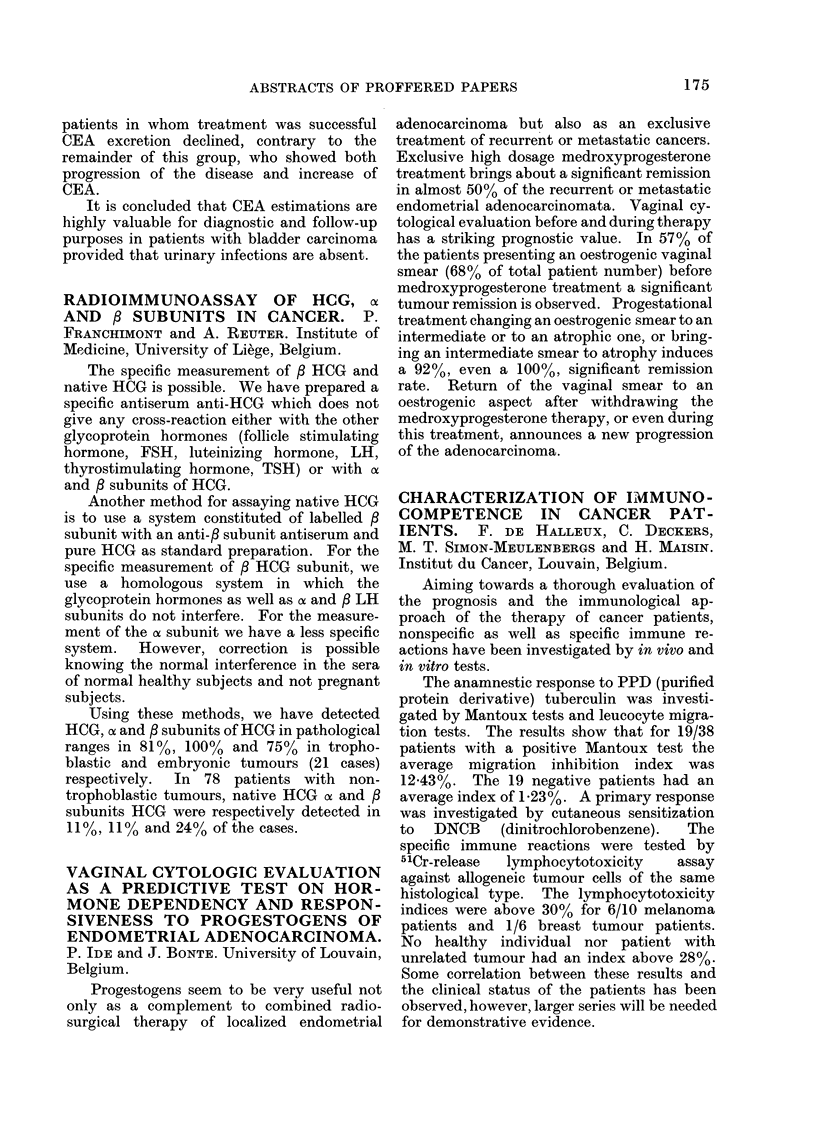# Proceedings: Vaginal cytologic evaluation as a predictive test on hormone dependency and responsiveness to progestogens of endometrial adenocarcinoma.

**DOI:** 10.1038/bjc.1974.139

**Published:** 1974-08

**Authors:** P. Ide, J. Bonte


### VAGINAL CYTOLOGIC EVALUATION AS A PREDICTIVE TEST ON HORMONE DEPENDENCY AND RESPONSIVENESS TO PROGESTOGENS OF ENDOMETRIAL ADENOCARCINOMA.

P. Ide and J. Bonte. University of Louvain, Belgium.

Progestogens seem to be very useful not only as a complement to combined radio-surgical therapy of localized endometrial adenocarcinoma but also as an exclusive treatment of recurrent or metastatic cancers. Exclusive high dosage medroxyprogesterone treatment brings about a significant remission in almost 50% of the recurrent or metastatic endometrial adenocarcinomata. Vaginal cytological evaluation before and during therapy has a striking prognostic value. In 57% of the patients presenting an oestrogenic vaginal smear (68% of total patient number) before medroxyprogesterone treatment a significant tumour remission is observed. Progestational treatment changing an oestrogenic smear to an intermediate or to an atrophic one, or bringing an intermediate smear to atrophy induces a 92%, even a 100%, significant remission rate. Return of the vaginal smear to an oestrogenic aspect after withdrawing the medroxyprogesterone therapy, or even during this treatment, announces a new progression of the adenocarcinoma.